# Intestinal *Klebsiella pneumoniae* infection enhances susceptibility to epileptic seizure which can be reduced by microglia activation

**DOI:** 10.1038/s41420-021-00559-0

**Published:** 2021-07-07

**Authors:** Peijia Lin, Aolei Lin, Kaiyan Tao, Min Yang, Qinglin Ye, Hongnian Chen, Yuanyuan Chen, Yuanlin Ma, Zijun Lin, Miaoqing He, Xuefeng Wang, Xin Tian

**Affiliations:** Department of Neurology, the First Affiliated Hospital of Chongqing Medical University, Chongqing Key Laboratory of Neurology, Chongqing, China

**Keywords:** Neuroscience, Epilepsy

## Abstract

Epilepsy is a common nervous system disease, and the existing theory does not fully clarify its pathogenesis. Recent research suggests that intestinal microbes may be involved in the development of epilepsy, but which microbe is involved remains unclear. We used 16s rRNA sequencing to identify the most relevant gut microbe. To determine the relationship between this microbe and epilepsy, we used an animal model. In addition, western blotting and immunofluorescence, as well as inhibitor studies, were used to evaluate and confirm the role of microglia in this process. In this study, we first report an increase in gut *Klebsiella pneumoniae* in patients with epilepsy. Subsequently, animal studies revealed that *Klebsiella pneumoniae* in the intestinal tract affects seizure susceptibility and activates microglial cells to release inflammatory factors. Furthermore, the inflammatory response of microglial cells plays a protective role in the seizure susceptibility caused by an increased abundance of *Klebsiella pneumoniae*. Our results suggest that gut disruption may be involved in seizure regulation and microglia protect the brain against seizure under this condition. These findings provide a new perspective for research on the pathogenesis and prevention of epilepsy.

## Introduction

Epilepsy is a public health problem in many clinical fields. Many diseases cause epileptic seizures, including those associated with internal medicine, external medicine, gynaecology, paediatrics, and infectious diseases [1]. Although the mortality rate of patients with epilepsy is several times higher than that of healthy individuals, many patients have achieved final remission in long-term seizures, indicating an endogenous anti-epileptic system in epilepsy patients that can fight against seizures [2, 3]. Thus, this endogenous anti-epileptic system may provide new insights into the prevention and treatment of epilepsy.

The discovery of the gut–brain axis provides a new perspective for human understanding of the occurrence and development of diseases [4, 5]. Humans have found that many frequently occurring diseases are associated with changes in gut microbes [6, 7]. Imbalances in the gut can trigger inflammation; however, the inflammatory response is not always harmful to humans [8]. Presently, the inflammatory response can be regulated by different mechanisms, and understanding the role of the inflammatory response in disease may identify new methods to treat diseases.

In patients with epilepsy, changes in the abundance and diversity of intestinal microbes have been observed, but their effects on the brain have been rarely reported [9]. Thus, this study aimed to investigate the brain responses induced by intestinal microbial changes to provide a new perspective for preventing and treating epilepsy. First, we used 16s rRNA sequencing and agarose electrophoresis to depict the characteristic intestinal flora spectrum of epilepsy patients. Compared with healthy controls, *Klebsiella pneumoniae(K. pneumoniae)* was increased in patients with epilepsy. Next, we found that the increased *K. pneumoniae* promoted seizures and activated microglia in an animal model. Inhibition of inflammatory cytokines released by activated microglia worsened the seizures, suggesting that these inflammatory cytokines exhibited endogenous protection against epileptic seizures induced by increased *K. pneumoniae*.

## Results

### Gut microbe composition of epilepsy patients

**Fig. 1:**
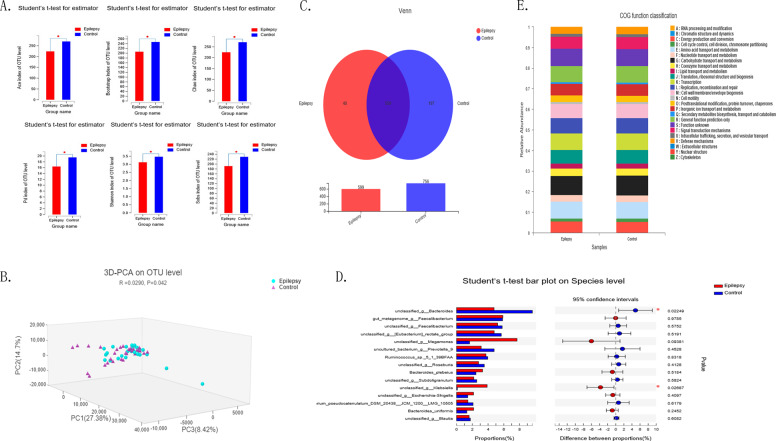
16s rRNA sequencing in the epilepsy and healthy groups. **A** Alpha diversity index (including ace, sobs, chao, bootstrap, pd, Shannon) showing a significant difference between the epilepsy group and healthy group (*n* = 30 in each group; *P < 0.05 epilepsy versus controls, Student’s *t* test). **B** Principal coordinate analysis (PCoA) of the sample was calculated using the Adnois algorithm, and the epilepsy group was significantly different from healthy population (*n* = 30 in each group; *P < 0.05 epilepsy versus controls). **C** The Venn diagram based on the OTU level shows that the epilepsy group had 40 specific OTUs. **D** *Bacteroides* and *Klebsiella* were significantly different between the epilepsy and control groups at the species level (*n* = 30 in each group; *P < 0.05 epilepsy versus controls, Student’s t test). **E** Bar chart depicting the gene ontology classification in epilepsy and control groups (*n* = 30 in each group).

The characteristics of the patients are summarised in Table 1. The gender distribution, age, and BMI are similar in two groups. When including epileptic patients, we choose patients who never used the anti-epileptic drug (AEDs) or who stopped using AEDs for at least 2 years. Hence, the time that they have been the diagnosis with epilepsy is variable, and the longest is about 14 years. Because some patients who were without AEDS for a long time but relapsed recently were also brought into study. The alpha diversity indexes (including ace, sobs, chao, bootstrap, pd, Shannon) of the epilepsy group based on the operational taxonomic unit (OUT) level were significantly lower than those of the healthy group (Fig. 1A). Principal coordinate analysis at the OUT level of the sample was calculated using the Adnois algorithm, and it was found that the microbial profile of epilepsy group was significantly different from healthy population (Fig. 1B). Species composition analysis showed that 559 operational taxonomic units were common between epilepsy patients and the healthy population, while the healthy population had 197 unique characteristic operational taxonomic units, which was significantly higher than the number unique characteristic operational taxonomic units for epilepsy patients (Fig. 1C). The above results suggest that the richness and diversity of intestinal microbes in epilepsy patients are significantly lower than those in the healthy population, which means that the microbial composition of faeces from epilepsy patients is significantly different from that of faeces from the healthy population. Next, we analysed the differences between epilepsy patients and the healthy population and found that the abundance of *Proteobacteria* in epilepsy patients was significantly higher than that in healthy people. At the species level, there were significant differences between the two groups in *Klebsiella* (Fig. 1D). Using PICRUSt to annotate the function of OTUs, and found that the COG function classification is similar between the two group (Fig. 1E).

**Table 1:** The characteristics of the patients and controls.

	Epilepsy (*N* = 30)	Control (*N* = 30)	*P* value
Average age	27.133	28.400	0.189
Female (*N*, %)	13 (43.33%)	13 (43.33%)	1.000
Average BMI (SD)	21.2600 (2.0)	21.2520 (2.0)	0.583
How long have been diagnosised	About 3 years (7days to 14 years)	–	
Seizure type	100%GTCS	–	
Egg abnormity	33.33%	–	
MRI abnormity	23.33%	–	

*BMI* body mass index, *GTCS* generalised tonic clonic seizure, *EGG* electroencephalogram, *MRI* magnetic resonance imaging.

Then, we used agarose electrophoresis to investigate what species of *Klebsiella* was significantly enriched in epilepsy patients and found that the abundance of *K. pneumoniae* was significantly enriched in patients with epilepsy (Supplementary Fig. 1A), while the abundances of *Klebsiella oxytoca (K. oxytoca*) in epilepsy patients and in healthy people showed no significant difference (Supplementary Fig. 1B). In summary, *K. pneumoniae* abundance was significantly increased in the gut of epilepsy patients, so it is speculated that *K. pneumoniae* is a key intestinal pathogen related to epilepsy.

### *Klebsiella pneumoniae* in the intestine promotes seizures and activates microglia

**Fig. 2:**
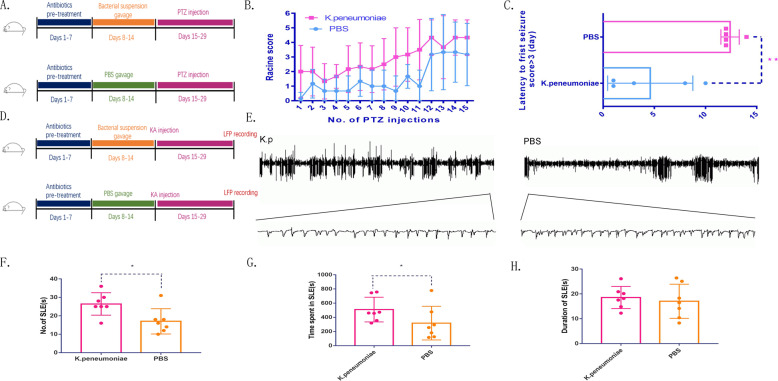
*K. pneumoniae* increases seizure susceptibility. **A** Graphic representation of the intervention timeline in the PTZ experiment. **B** In the PTZ kindling mouse model, the seizure score was higher in the *K. pneumoniae* group than in the PBS group after the PTZ injection (*n* = 6 in each group. Error bars represent the means ± SEMs; *P < 0.05, ANOVA). **C** *K. pneumoniae* group mice exhibited a significantly shortened latency time to the first seizure, scoring > 3 (**P < 0.01, *n* = 6 in each group, Student’s *t*-tests). **D** Graphic representation of the intervention timeline in the KA experiment. **E** Representative LFPs in the two groups. **F** During 30 min of recodring, the number of SLEs was significantly higher in the *K. pneumoniae* group than in the PBS group (*P < 0.05, *n* = 7 in each group, Student’s *t* test). **G** The total time spent in SLEs during the 30 min was significantly higher in the *K. pneumoniae* group than in the PBS group (*P < 0.05, *n* = 7 in each group, Student’s *t* test). **H** The duration of SLEs was not significantly changed between the groups.

Changes in the abundance of *K. pneumoniae* in the intestine may represent a correlation or causality with epilepsy. Next, we used animal models to test whether the abundance of *K. pneumoniae* in the gut affected seizures. All mice were first pretreated with antibiotics. After the completion of pretreatment, the experimental group was gavaged with a *K. pneumoniae* suspension for 7 consecutive days to increase the abundance of *K. pneumoniae* in the intestine, while the control group was gavaged with sterile PBS as the control group (Supplementary Fig. 2). Mice in both groups were injected simultaneously with pentylenetetrazol (PTZ) for 15 consecutive days to build the classic epilepsy model (Fig. 2A). On the first day of the study, there were grade 5 seizures in the experimental group, while there was only one grade 1 seizure in the control group. As the number of PTZ injections increased, the level of seizures in both groups gradually increased. The experimental group showed a higher intensity of seizures than the control group during this 15 days (Fig. 2B). The mean latent period was 4.600 ± 1.860 days in the experimental group and 12.40 ± 0.4000 days in the control group. Therefore, there was a significantly shorter mean latent period in the experimental group than in the control group (Fig. 2C). The same intervention was also given to both groups of mice before using kainic acid (KA) to induce SE, and the local field potential (LFP) of the mice was recorded 15 days after SE (Fig. 2D). Similar to previous reports, seizure-like events were observed in both groups of mice (Fig. 2E). In the experimental group, the frequency of seizure-like events and the time spent in seizure-like events was significantly higher than those in the control group (Fig. 2F–G). However, the difference in the average duration of seizure-like events between the two groups was not significant (Fig. 2H). In summary, the increase in *K. pneumoniae* abundance in the intestine facilitated seizures.

To further investigate how *K. pneumoniae* is associated with epilepsy, we next explored whether *K. pneumoniae* in the intestine has a communication pathway with the brain. Using Fluorescein isothiocyanate (FITC) to label K*. pneumoniae*, we found that *K. pneumoniae* in the intestine may have some connection with the brain. After *K. pneumoniae* was labelled with FITC, green fluorescence was observed. Twelve hours after gavage of the FITC-labelled *K. pneumoniae* suspension, the colon and brain tissues of the mice were taken for fluorescence detection. Both the colon and brain displayed detectable green fluorescence (Supplementary Fig. 3A–C).

**Fig. 3:**
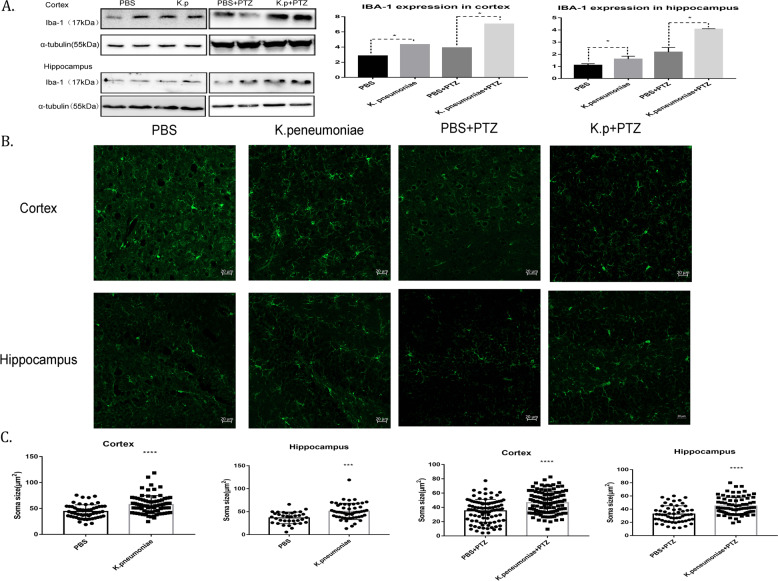
*K. pneumoniae* activates microglia in mice. **A** Representative images of Iba-1 expression in the cortex and hippocaumps before and after the PTZ test. Compared with that in the controls, Iba-1 expression was consistently increased in the cortexes and hippocaumps of mice who were administered *K. pneumoniae* via gavage in two groups (*n* = 3 in each group; *P < 0.05 versus controls, Student’s *t* test). **B**, **C** Representative confocal images of microglia; cell body changes in the cortexes and hippocampi of mice on the 14th and 29th days and a summary of the results (PBS, cortex, *n* = 57; *K. pneumoniae*, cortex, *n* = 78; PBS + PTZ, cortex, *n* = 93; *K. p* + PTZ, cortex, *n* = 114; PBS, hippocampus, *n* = 35; *K. pneumoniae*, hippocampus, n = 53; PBS + PTZ, hippocampus, *n* = 56; *K. p* + PTZ, hippocampus, *n* = 76; ***P < 0.001, ****P < 0.0001, Student’s *t* test).

**Fig. 4:**
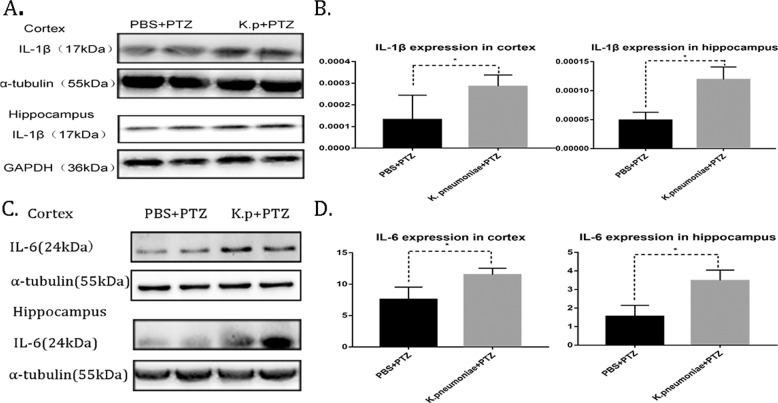
*K. pneumoniae* activates microglia mediated inflammatory response in mice. **A**, **B** Representative images and the quantification of IL-1β expression in the cortex and hippocaumps after PTZ injection. Compared with that in the controls, IL-1β expression was increased in the cortex and hippocampus in the *K. pneumoniae* group (*n* = 3 in each group; *P < 0.05, Student’s *t* test). **C**, **D** Representative images and the quantification of IL-6 expression in the cortex and hippocaumps after PTZ injection. Compared with that in the controls, IL-6 expression was increased in the cortex and hippocampus in the *K. pneumoniae* group (*n* = 3 in each group; *P < 0.05, Student’s *t* test).

Considering that *K. pneumoniae* in the gut may communicate with the brain and that glial cells are an important central immune barrier, we therefore used western blotting to detect the expression of a microglia activation marker (Iba-1). The results showed that throughout the whole period of PTZ kindling, the experimental group had significantly increased expression of Iba-1 in the cortex and hippocampus (Fig. 3A). Then, we used immunofluorescence to explore whether *K. pneumoniae* can cause morphological activation of microglia. We found that the increase in *K. pneumoniae* significantly augmented microglial cell bodies in the hippocampus and cortex throughout the behavioural test (Fig. 3B–C). These results suggest that increased an abundance of *K. pneumoniae* can significantly activate microglia. Subsequently, the expression of the IL-1β and IL-6, which are two main inflammatory markers of activated microglia, were detected. And it was found that the expression of IL-6 and mature IL-1β in the brain of the experimental group significantly increased throughout the behavioural test (Fig. 4A–D). These results suggested that the increased abundance of *K. pneumoniae* in the intestine activated microglial cells and promoted the release of corresponding inflammatory factors in an epilepsy animal model.

### Activated microglial cells release its downstream inflammatory factors to protect against seizures

**Fig. 5:**
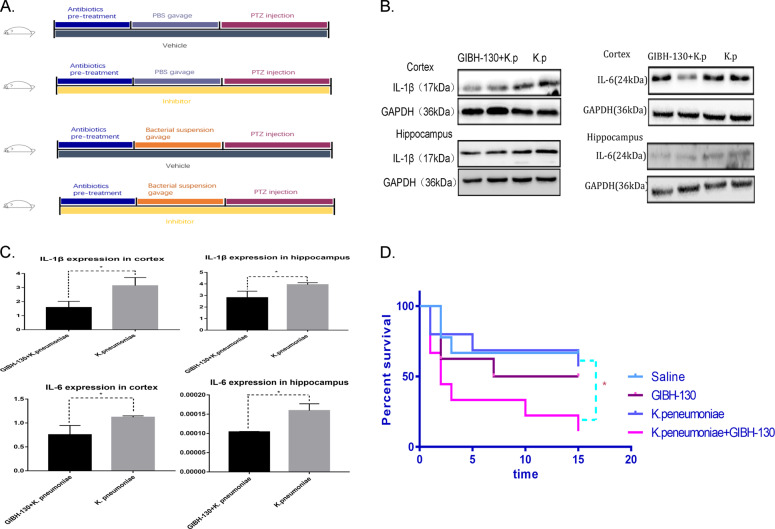
Inhibiting the release of cytokines by activated microglial cells aggravates seizures. **A** Graphic representation of the intervention timeline in the animal experiment. **B** Representative images of IL-1β and IL-6 expression in the cortex and hippocaumps of mice treated with *K. pneumoniae* and GIBH-130 + *K. pneumoniae* respectively. **C** Quantification of IL-1β and IL-6 expression in the cortex and hippocaumps in two groups. Compared with that in the *K. pneumoniae* group, IL-1β and IL-6 expression was decreased in the cortex and hippocaumps of mice in the GIBH-130 + *K. pneumoniae* group (*n* = 3 in each group; *P < 0.05 versus controls, Student’s *t* test). **D** Percentage survival over the number of PTZ injections (*n* = 8 in each group; *P < 0.05, log-rank test).

Increased abundance of *K. pneumoniae* in the intestine not only facilitates seizure but also activates microglia to release inflammatory cytokines. However, it is not clear how the microglial cell-mediated inflammatory response influence seizures. Therefore, GIBH-130, which has been verified to inhibit the production of IL-1β and IL-6 by activated microglial cells, was used for intervention (Fig. 5A) [10]. And it was found that when the process of releasing inflammatory foctors by activated microglial cells was inhibited (Fig. 5B, C), the severity of epileptic seizures in mice with increased *K. pneumoniae* abundance was aggravated. When mice were given GIBH-130 and the *K. pneumoniae* suspension at the same time, their mortality in the PTZ kindling model was significantly higher than that of mice given only the *K. pneumoniae* suspension (*p* < 0.05), while the mortality of mice in the GIBH-130 group and vehicle group was similar to that of mice in the *K. pneumoniae* alone group (Fig. 5D). This finding suggests that when the abundance of *K. pneumoniae* increased in the intestine, microglial cells were activated and released corresponding inflammatory factors, and this activated microglial cell-mediated inflammatory response reduced the influence of *K. pneumoniae* on seizures. In summary, *K. pneumoniae* in the intestine promotes seizures, and activated microglial cells protect brain under this condition.

## Discussion

In this study, we found for the first time that *K. pneumoniae* was increased in the gut of epileptic patients and that the abundance of *K. pneumoniae* in the intestine could affect seizure susceptibility in animal models of epilepsy. In addition, we found that increased abundance of *K. pneumoniae* in the intestine activated microglia. The inflammatory response mediated by activated microglia can attenuate the effect of increased *K. pneumoniae* on seizure susceptibility to some extent.

In 2018, Peng et al. [11] explored the difference between drug-sensitive epilepsy patients and drug-resistant epilepsy patients by 16s rRNA sequencing, and found that drug-sensitive epilepsy patient showed significantly increase of *bifidobacterium* and *lactobacillus* when compared with drug-resistant epilepsy patients, suggesting that abundance of *bifidobacterium* and *lactobacillus* may be associated with drug resistance of AEDs. In 2019, Dahlin et al. [9] investigated the intestinal microbes of 30 epilepsy patients who were diagnosed with idiopathic focal epilepsy and ten healthy controls, and found that *Proteobacteria phylum* was higher in patients with epilepsy. However, this study did not mention the medication condition of the epilepsy patients they included, so we can hardly rule out the effect of AEDs on intestinal microbes. In our study, the patients that we included are those who had never taken AEDs or had not taken AEDs in recent 2 years, so as to avoid the influence of AEDs on the intestinal microbes and find the most relevant intestinal bacteria related to the occurrence and development of epilepsy. And also, the patients we enroled were all characterised as generalised tonic–clonic seizure, which is different from Dahlin et al. [9].

In the faecal samples of epilepsy patients, we found a significant increase in the abundance of *Klebsiella* species, which is similar to the results detected by Lin et al. [10] in patients with depression. Maes et al. [12] found that when depressed patients were exposed to *K. pneumoniae* LPS, their IgA levels in their blood increased significantly, so they speculated that *K. pneumoniae* translocation might be involved in the development of major depression. *K. pneumoniae* and *K. oxytoca* exist in the human intestinal tract, and they are the major pathogenic *Klebsiella* species. Therefore, their abundance in faecal samples was further verified in our study.

To understand the relationship between *K. pneumoniae* and epilepsy, we selected the epilepsy model for verification. In order to avoid false-negative and false-positive results, we used PTZ and KA epilepsy models. Considering that epileptic patients are not completely sterile individuals, to better simulate the pathophysiological process of epileptic patients, we chose specific-pathogen-free mice instead of germ-free mice for behavioural verification. In animal studies, PBS group was setted to rule out the effect of injury by gavage. So, when compared the two group, we can know precisely about the effect of *K. pneumoniae*. However, it could better if there is another control group, which is treated with a common intestinal colonising microbe. According to Olson et al. [13], *bifidobacterium longum* had no treatment effect in 6-Hz seizure model, which is a model of refractory epilepsy. In PTZ model, both Eor et al. [14] and Bagheri et al. [15] found that probiotic bacteria can reduce the severity of seizures. So, gavage some probiotic bacteria may have some benefit effect in PTZ model.

FITC was used to label *K. pneumoniae*, and it was found that *K. pneumoniae* in the intestinal tract may communicate with the brain, which was consistent with a report of conference in Science (https://www.sciencemag.org/news/2018/11/do-gut-bacteria-make-second-home-our-brains). However, this study hasn’t been officially published yet. And this FITC-labelled *K. pneumoniae* somehow has some limitations, and it would be better if the *K. pneumoniae* show green fluorescence stably. One might think may be that *K. pneumoniae* was killed and phagocytosed by antigen-presenting cells in the gut that later trafficked to the brain.

Microglia are macrophages in the central nervous system, which play a key role in maintaining brain homoeostasis, and increasing evidence shows that gut microbes can affect the maturation and function of microglia [16, 17]. We then examined markers of microglial activation and found that microglia were activated in the cortex and hippocampus and that inflammatory cytokines were increased when *K. pneumoniae* abundance increased in the intestine.

To further understand how the microglial activation-mediated inflammatory response influence the regulation of seizure susceptibility in this condition, we used GIBH-130 to inhibit microglial activation of inflammation. When the activated microglial cell-mediated inflammatory reaction was inhibited, 7 of 8 mice with increased gut *K. pneumoniae* died in the process of generalised tonic–clonic seizures. In the PTZ kindling model, death during generalised tonic–clonic seizures represents a more severe seizure grade [18]. It has been previously believed that the release of inflammatory cytokines after microglial activation can promote the development of epilepsy [19]. However, our data suggest a contrary conclusion: the inflammatory response mediated by activation of microglial cells in these mice did not promote the development of epilepsy, and on the contrary, it could reduce the influence of increased gut *K. pneumoniae* abundance on seizure susceptibility to some extent. Waltl et al. [20] found in an animal model of viral encephalitis that the removal of microglia accelerated the occurrence of seizures, and they believed that microglia played a protective role in the seizures induced by viral encephalitis. The results of Fekete et al. [21] suggested that selective clearance of microglia expanded the area of viral infection and significantly increased the flow of virus particles into the brain parenchyma. Therefore, we hypothesise that when the gut *K. pneumoniae* abundance increases, *K. pneumoniae* may somehow communicate with the brain and activate microglia to release a series of inflammatory factors. When the inflammatory response is inhibited, seizures aggravated. Microglia may be the key to seize seizure when the imbalance of intestinal microbes occurs. However, GIBH-130, the microglia inhibitor we used, can only inhibit the inflammatory response of activated microglia.The effect of non-inflammatory pathway is still under exploration.

In general, our study demonstrated an increased abundance of *K. pneumoniae* in the intestine of epileptic patients and demonstrated that the increased abundance of *K. pneumoniae* in the intestine could increase seizure susceptibility in animal models. We found that microglial cell-mediated activation of inflammatory responses protected against seizures in mice with increased intestinal *K. pneumoniae*. These findings provide a new perspective for research on the pathogenesis and prevention of epilepsy.

## Materials and methods

### Selection of patients

This study was approved by the Chongqing Medical University, and all the participants were informed. In total, 30 epilepsy patients and 30 healthy controls were included. The inclusion and exclusion criteria are shown in Table 2.

**Table 2:** The inclusion and exclusion criteria.

Epilpsy group	Healthy group
1. Met the diagnostic criteria for epilepsy.	1. No organic diseases or mental diseases.
2. Did not take anti-epileptic drugs for nearly two years and did not take antibiotics or probiotics for nearly half a year.	2. No history of drug abuse; did not take antibiotics or prebiotics for half a year.
3. Eighteen to sixty years old.	3. Eighteen to sixty years old.
4. Without other brain, heart, liver, or kidney diseases, diabetes or other serious physical and mental diseases.	4. Normal laboratory routine examination.
5. Normal laboratory routine examination.	5. Excluded female patients who were breastfeeding, menstruating, or pregnant
6. Excluded female patients who were breastfeeding, menstruating, or pregnant.	

### Faecal sample collection and DNA extraction

All the subjects were required to defecate on an empty stomach in the morning, and the samples were maintained on ice during transportation. Next, the samples were frozen in liquid nitrogen and stored at −80 °C. An E.Z.N.A.^®^ soil DNA kit (Omega Bio-tek, Norcross, GA, USA) was used to extract total faecal DNA. The entire process was performed in a biosafety cabinet. After extraction, the genomic DNA concentration and purity in each sample were determined using a Nano-Drop 2000 spectrophotometer (Thermo Scientific, MA, USA), and the DNA extraction quality was detected by 1% agarose gel electrophoresis.

### 16S rRNA sequencing

The V3–V4 variable region was amplified using the 338F (5′-ACTCCTACGGGAGGCAGCAG-3′) and 806R (5′-GGACTACHVGGGTWTCTAAT-3′) primers. The amplification procedure was as follows: pre-denaturation at 95 °C for 3 min; 27 cycles of denaturation at 95 °C for 30 s, annealing at 55 °C for 30 s, and extension at 72°C for 30 s; and extension at 72°C for 10 min (instrument: ABI GeneAmp^®^ 9700). The amplification system was assembled in a volume of 20 µl comprising 4 µl of 5*FastPfu buffer, 2 µl of 2.5 mm dNTPs, 0.8 µl of primer (5 µM), 0.4 µl of FastPfu polymerase, and 10 ng of DNA template. The polymerase chain reaction (PCR) products were separated using a 2% agarose gel, purified using an AxyPrep DNA Gel Extraction Kit (Axygen Biosciences, Union City, CA, USA), eluted with Tris-HCl, and detected by 2% agarose electrophoresis. QuantiFluor™-st (Promega, USA) was used for quantitative assays. PE 2 × 300 libraries were constructed from purified amplified fragments according to the standard operating procedures of the Illumina MiSeq platform (Illumina, San Diego, USA) and then were sequenced using Illumina’s MiSeq PE300 platform.

### PCR amplification and agarose electrophoresis

Primers for *K. pneumoniae* and *K. oxytoca* were used to amplify the DNA of *K. pneumoniae* and *K. oxytoca* in stool samples (the upstream and downstream primers were as follows: *K. pneumoniae*:5′-TGATTGCATTCGCCACTGG-3′, 5′-GGTCAACCCAACGATCCTG-3′: 486 bp; *K. oxytoca*: 5′-GGACTACGCCGTCTATCGTCAAG-3′, 5′-CACCGTAAAGGCATACTCCGTATC-3′: 193 bp) [22, 23]. The amplification system was assembled in a volume of 50 µl (5 ng of DNA, 0.4 µl of upstream and downstream primers, 25 µl of Taq enzyme, and deionised water balanced to 50 µl). A PCR instrument (Bio-Rad, T100) was used for amplification. The reactions were conducted according to the instructions for the Taq enzyme (Vazyme, Nanjing, China, P112-01). To determine the abundance of *K. pneumonia* and *K. oxytoca*, the amplified products were visualised by 2% agarose electrophoresis.

### Fluorescein isothiocyanate isomer-labelled bacteria

A clinical strain of *K. pneumoniae* (CMCC46117) was purchased from the Guangdong Culture Collection Centre (China). The bacteria were grown in liquid Luria–Bertani medium for 12 h (37 °C, 150 rpm), the culture was centrifuged for 10 min (4 °C, 5000 × *g*), the supernatant was discarded, and the pellet was washed twice with sterile phosphate buffer saline (PBS). Next, the bacteria were re-suspended in a solution of FITC isomer (1 mg/ml) diluted with sterile PBS and incubated at 37 °C for 2 h in the dark. After incubation, the labelled bacteria were washed with sterile PBS solution 6 times to remove unbound FITC, and the FITC-labelled *K. pneumoniae* suspension was finally suspended with sterile PBS to achieve a concentration of 5 × 10^9^ cfu/ml. The mice were given 1 ml of FITC-labelled *K. pneumoniae* suspension by gavage and then were sacrificed 12 h later [24].

### Animals

The adult C57BL/6 mice (6–8 weeks old) used in this study were provided by the Experimental Animal Centre of Chongqing Medical University. All animal experiment procedures strictly complied with animal ethics requirements and were approved by the animal ethics committee of Chongqing Medical University. All the mice were raised in a specific-pathogen-free environment using a standard method.

### Antibiotic intervention

In accordance with the method previously described by Reikvam et al. [25], the mice were given 50 mg/kg of vancomycin (Macklin, China), 100 mg/kg of neomycin (Solarbio, China), and 100 mg/kg of metronidazole (Macklin, China) every 12 h for a total of 7 consecutive days, and 1 mg/ml of ampicillin (Macklin, China) was added to the drinking water at the same time.

### Bacterial suspension intervention

The bacteria were grown in liquid Luria–Bertani medium for 12 h (37 °C, 150 rpm), centrifuged and washed, and the concentration reached 5 × 10^9^ cfu/ml by re-suspension with sterilised PBS. The experimental group was given 200 µl of bacterial suspension every 12 h for 7 consecutive days, while the control group was given 200 µl of sterile PBS. The DNA of faecal samples was obtained after the bacterial intervention and at the end of the epilepsy behaviour test to ensure the efficacy of the intervention.

### Pentylenetetrazol kindling model

Mice were intraperitoneally injected with 35 mg/kg of PTZ (Sigma-Aldrich Co., St. Louis, USA) once daily for 15 days. After each intraperitoneal injection of PTZ, the mice were placed in a transparent box to observe seizures for 1 h. The grade of seizure was scored according to the Racine scale [26].

### Kainic acid model and field potential recording

After anaesthesia, the mice were fixed on a stereotaxic instrument (RWD Life Science Co., Ltd, Shenzhen, China). The head hair was trimmed to expose the top of the skull of the mice. The skin of the surgical area was disinfected with iodophor, the scalp was cut open under sterile conditions, the hypodermic fascia was disinfected with hydrogen peroxide, and the skull was fully exposed. Using a 0.5-µl syringe (Hamilton, Reno, NV), 50 nl of saline containing 1.0 nmol of KA (Sigma-Aldrich Co., St. Louis, USA) was injected into the right hippocampus, and the whole injection time lasted 3 min. The syringe was held still for 5 min to reduce reflux along the injection trajectory. The LFP was recorded after 15 days. Before recording the LFP, a multichannel microwire array (platinum–iridium alloy wires; 25-μm in diameter; Plexon, Dallas, TX, USA) was implanted in the right dorsal hippocampus. The head of the awake mice was fixed to minimise changes in LFPs caused by behavioural states. LFPs were recorded using the MAP data acquisition system (Plexon, Dallas, TX). The data were inspected by NeuroExplorer (Nex Technologies, Littleton, MA).

### Western blotting

Mouse brain tissue samples were collected for western blot analysis using 10–15% SDS-polyacrylamide gels to resolve protein samples. The gels were then electrophoretically transferred to 0.45-mm polyvinylidene difluoride membranes (Millipore, Billerica, MA, USA). After blocking with 8% milk in Tris-buffered saline with Tween (TBST) at room temperature for 1 h, rabbit anti-Iba-1 (1:1000; Wako, Japan), rabbit anti-IL-1β (1:500; Wanleibio, China), rabbit anti-IL-6(1:1000; Proteintech, China), mouse anti-GAPDH (1:10000; Proteintech, China) or mouse anti-α-tubulin (1:3000; Invitrogen, USA) primary antibodies were incubated with the membranes overnight at 4 °C. On the second day, the membranes were washed in TBST and then incubated with horseradish peroxidase-conjugated secondary antibodies for 1 h (1:5000; Proteintech, China). TBST was then used to wash the membrane three times (10 min each time). The membranes were placed on a gel imager, and enhanced chemiluminescence reagent (Thermo, Marina, CA, USA) was added to visualise the bands.

### Immunofluorescence

The tissue sample was fixed with 4% paraformaldehyde overnight (4 °C) and then dehydrated in a 30% sucrose solution for 48 h (4 °C). The tissue sample was then embedded with optimal cutting temperature compound, placed into isopentane precooled to −80 °C for 10 s, and finally stored at −80 °C. The tissue sample was cut into 20-µm slices, incubated with 0.4% Triton X-100 to disrupt the membrane for 30 min, and blocked with goat serum (Boster, Wuhan, China) for 1 h. The sections were incubated at 4 °C overnight with the primary antibodies rabbit anti-Iba-1 (1:500; Wako, Japan). The unbound primary antibody was washed with PBS on the second day, and the tissue sample was incubated with a secondary antibody for 1 h at RT in the dark. Then washed the slides, and made the slides dry. The antifade mounting medium (including DAPI) was dripped onto the slides, and covered with glass for storage. Confocal laser scanning microscopy (Leica, Wetzlar, Germany) was used to capture images. Fluorescence data were analysed using ImageJ.

### Inhibitor intervention

The mice were orally administered the inhibitor GIBH-130 (MCE; hy-101860; 0.25 mg/kg) at the beginning of the intervention and then once a day until the end of the behaviour test. The control group mice received oral saline gavage once a day until the end of the behaviour test [27].

### Statistical analysis

SPSS (version 22.0; SPSS Inc., Chicago, IL, USA) was used for statistical analyses, and GraphPad was used to construct the figures. The 16s rRNA sequence data were analysed using the online Majorbio Cloud Platform based on Qiime. The onset level of PTZ behaviour was tested by repeated chi-square analysis, and the survival curve was plotted using the Kaplan–Meier method. The latent period in the PTZ model, KA model data, and real-time PCR, western blotting, and immunofluorescence results were analysed using nonpaired *t* test.

## Supplementary information

supplemental figure1

supplemental figure 2

supplemental figure 3

supplement legend

## Data Availability

The datasets generated and/or analysed during the current study are not publicly available, but are available from the corresponding author on reasonable request.
