# Supplementary figures and images for: Intestinal Klebsiella pneumoniae infection enhances susceptibility to epileptic seizure which can be reduced by microglia activation
Source: Cell Death Discov. 2021 Jul 7;7:175. doi: 10.1038/s41420-021-00559-0 (PMC8263697; doi:10.1038/s41420-021-00559-0)

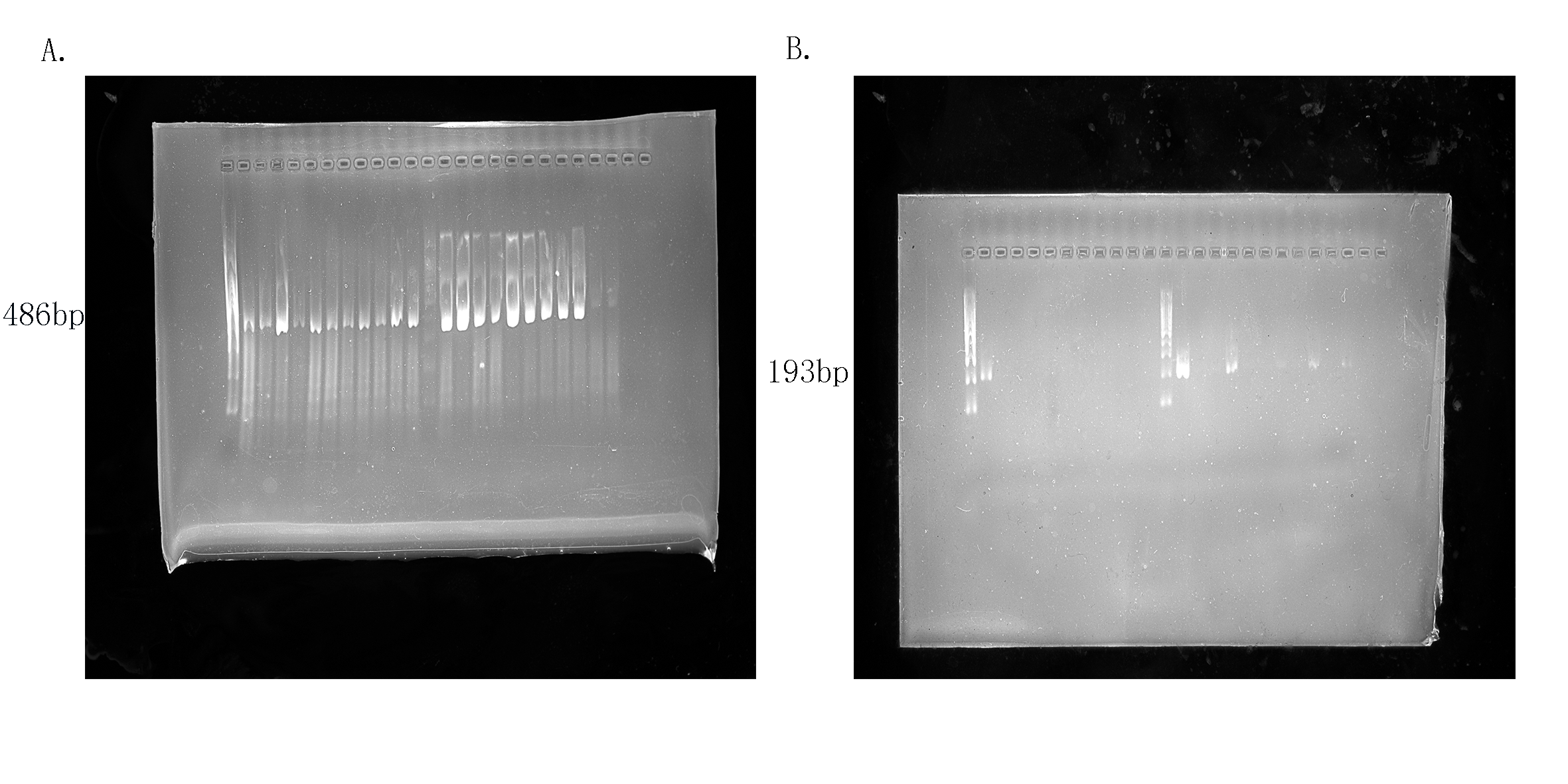

Supplement: Supplementary file 1 — supplemental figure1 [file 41420_2021_559_MOESM1_ESM.tif]

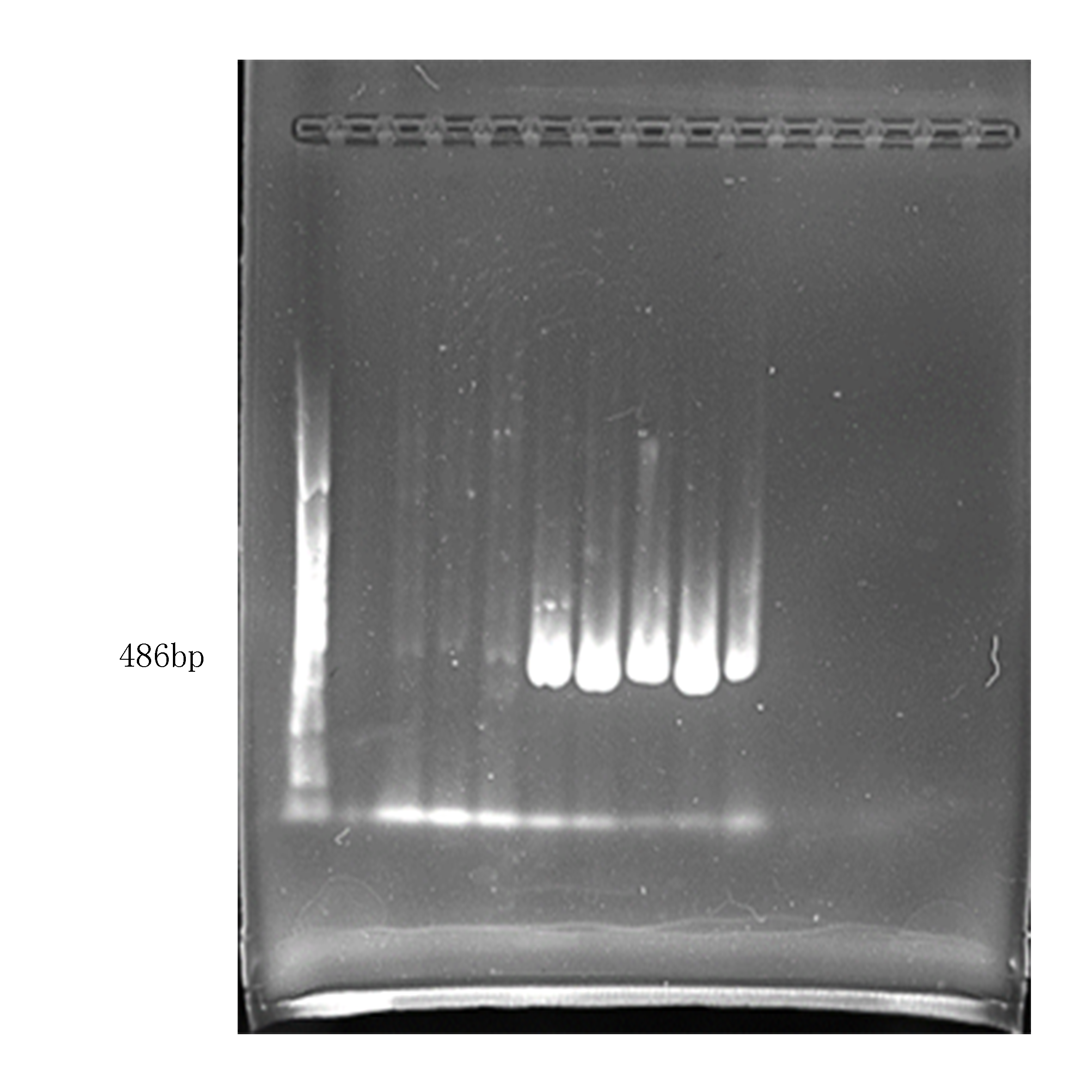

Supplement: Supplementary file 2 — supplemental figure 2 [file 41420_2021_559_MOESM2_ESM.tif]

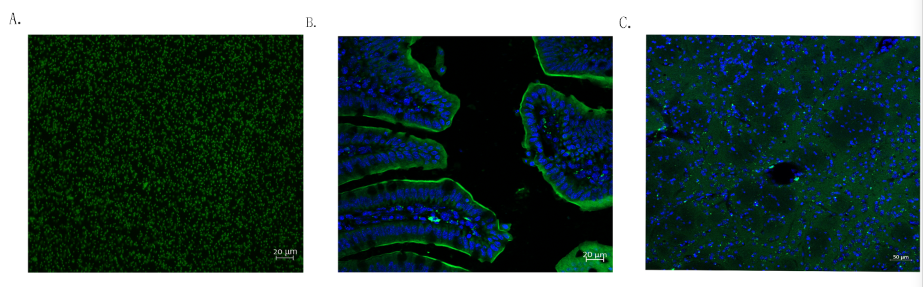

Supplement: Supplementary file 3 — supplemental figure 3 [file 41420_2021_559_MOESM3_ESM.tif]
