# Supplementary material for: Intestinal Klebsiella pneumoniae infection enhances susceptibility to epileptic seizure which can be reduced by microglia activation
Source: Cell Death Discov. 2021 Jul 7;7:175. doi: 10.1038/s41420-021-00559-0 (PMC8263697; doi:10.1038/s41420-021-00559-0)
Supplement: Supplementary file 4 — supplement legend [file 41420_2021_559_MOESM4_ESM.docx]

**Supplement 1. *K. pneumoniae* may be the potential pathogen in the gut of epilepsy patients**. (A) The epilepsy group showed significantly increased *K. pneumoniae* expression (n = 11 in each group)*.* (B) There is no significant difference in *K. oxytoca* expression between the two groups(n = 11 in each group).

**Supplement 2. Intestinal *K. pneumoniae* may communicate with brain**

(A to C) Representative confocal images showing that *K. pneumoniae* were successfully labbelled with green fluorescence. After gavage administration of a FITC-labelled *K. pneumoniae* suspension, the green fluorescence of *K. pneumoniae* can be dected in the gut and brain simultanously.

**Supplement 3. Agarose electrophoresis to investigate the level of *K. pneumoniae* in the faeces of mice treated with PBS and *K. pneumoniae* after 7 days** . The first lane represents DNA ladder(100-1000bp)，the second to fifth lanes represent mice in the control group (PBS gavage), and the remaining lanes represent mice in the experimental group (*K. pneumoniae* suspension gavage). It was found that the abundance of *K. pneumoniae* was higher in the experimental group.
